# Overview of approved CAR‐T therapies, ongoing clinical trials, and its impact on clinical practice

**DOI:** 10.1002/jha2.338

**Published:** 2021-11-24

**Authors:** Salyka Sengsayadeth, Bipin N. Savani, Olalekan Oluwole, Bhagirathbhai Dholaria

**Affiliations:** ^1^ Vanderbilt Ingram Cancer Center Vanderbilt University Medical Center Nashville Tennessee USA

**Keywords:** acute lymphoblastic leukemia, CAR‐T cell therapy, diffuse large B‐cell lymphoma, multiple myeloma, non‐Hodgkin lymphoma

## Abstract

In recent years, we have seen rapid expansion of chimeric antigen receptor T‐cell (CAR‐T) therapies in multiple malignancies. CAR‐T therapy has profoundly altered the treatment landscape of non‐Hodgkin lymphoma, B‐cell acute lymphoblastic leukemia, and multiple myeloma. Currently available CD19 and B‐cell maturation antigen‐directed CAR‐T therapies have shown high overall response rate and durable remissions in patients who have failed standard therapies. Multiple studies are underway exploring the role of CAR‐T‐cell therapy as earlier line of treatment. In high‐grade B‐cell lymphoma, CD19 CAR‐T therapy may replace autologous hematopoietic cell transplantation as second line therapy in near future. CAR‐T‐cell therapy targeting novel tumor‐associated antigens will help expand utility of this treatment modality in other hematological malignancies. It may also help overcome limitations of currently approved CAR‐T‐cell therapies. In this review, we have provided an overview of currently approved CAR‐T therapies and upcoming clinical trials which may potentially impact the clinical practice.

## 1 OVERVIEW OF APPROVED CAR‐T THERAPIES

Chimeric antigen receptor T‐cell (CAR‐T) therapies are one of the most cutting‐edge cancer therapies available today. Developments in T cell technology harness an individual's own T cell's ability to be engineered to recognize tumor cell surface proteins and in turn cause cancer cell death has led to several United States Food and Drug Administration (FDA)‐approved CAR‐T cell therapies to patient with aggressive hematologic malignancies. As of October, 2021, there are four currently approved CAR‐T products available for non‐Hodgkin lymphomas (NHL), two for B‐cell acute lymphoblastic leukemia (B‐ALL) and one for multiple myeloma (MM). Many more are currently in the pipeline of clinical development for these and other malignancies.

### 1.1 NHL and B‐ALL

The currently available CAR‐T therapies for NHL utilize CD19 as a target. CD19 is a cell surface antigen that is expressed on malignant and normal B cells. CAR‐T products are engineered by transduction of CAR gene into healthy T cell with a viral vector. Subsequently, CAR‐T cells are expanded ex vivo to produce a CAR‐T product that is infused in patients with relapsed or refractory (R/R) high grade hematologic malignancies such as NHLs, B‐ALL, and mantle cell lymphoma (MCL). These engineered T cells bind to CD‐19 positive cells, this results in activation via CD28 or 4‐1BB costimulatory domain which results in activation and in vivo proliferation of CAR‐T cells that cause elimination of the CD19 positive malignant cells [1].

For R/R high‐grade B‐cell lymphoma (HGBCL), there are currently three CAR‐T cell products approved for patients who have received at least two lines of therapies: axicabtagene ciloleucel (axi‐cel), tisagenlecleucel (tisa‐cel), and lisocabtagene ciloleucel (liso‐cel). Brexucabtagene autoleucel (brexu‐cel) is approved for R/R MCL. Axi‐cel, tisa‐cel, liso‐cel, and brexu‐cel are genetically modified anti‐CD19 autologous CAR‐T‐cells that are designed to target CD19 in B‐cell malignancies, and all four have showed significant activity in poor risk R/R NHL as herein discussed. Tisa‐cel is also approved for relapsed B‐ALL in pediatric and young adult patients (≤25 years of age).

## 2 AXICABTAGENE CILOLEUCEL

The first FDA approved CAR‐T product was axi‐cel. The data that led to this initial FDA approval were based upon the ZUMA‐1 trial, which was a phase 2, multicenter trial that included 111 patients with R/R HGBCL, primary mediastinal B cell lymphoma, or transformed follicular lymphoma (FL). After receiving lymphodepleting chemotherapy with fludarabine and cyclophosphamide, patients received a single infusion of axi‐cel. Results from this trial showed high overall response rates (ORRs) of 82% with complete response (CR) rates of 54%. After the median follow‐up of 15.4 months, 40% of patients remained in remission. Overall survival (OS) was 52% at 18 months. Common CAR‐T toxicities such as cytokine release syndrome (CRS) and immune effector cell‐associated neurotoxicity syndrome (ICANS) were common. Grades 3 and higher CRS and ICANS were reported in 13% and 23% of patients, respectively [2]. Based on these data, axi‐cel became the first FDA CAR‐T product on October 18, 2017. Longer‐term follow‐up of the ZUMA‐1 trial result was published by Locke et al. with a median follow‐up of 27.1 months. The median duration of response was 11.1 months with the median progression free survival (PFS) of 5.9 months and OS not yet reached. CRS and ICANS were similar to previously reported in the initial ZUMA‐1 trial, and no new treatment‐related adverse events were subsequently reported. These follow‐up data confirmed that axicabtagene ciloleucel induced durable responses with a median OS greater than 2 years and a very well tolerated long‐term safety profile [3].

In the real‐life application of axi‐cel as standard of care (SOC), additional outcomes data from US Lymphoma CART consortium, which is a group of 17 US institutions, showed that SOC axi‐cel outcomes were comparable to trial data for R/R HGBCL. This retrospective study included 275 patients who received axi‐cel, despite many of these patients (43%) would not have met eligibility criteria of ZUMA‐1 trial to comorbidities. Best ORR and CR rates were 82% and 64%, respectively, with comparable rates of CRS and ICANS as in ZUMA‐1. Median PFS was 8.3 months, and OS had not yet been reached. They did discern that patients with Eastern Cooperative Oncology Group (ECOG) performance status of 2–4 and elevated LDH had reduced PFS and OS in this study but overall, the response rates, CR rates, and toxicity rates, particularly of CRS and ICANS were similar to the ZUMA‐1 trials [4].

Axi‐cel received its second indication on March 5, 2021, when it received accelerated FDA approval for patients with R/R FL after two or more lines of therapy. The approval was based on the ZUMA‐5 study (NCT03105336) which was a single arm, open label study of 81 patients with R/R FL or marginal zone lymphoma (MZL). The results showed an ORR of 92% with 76% of patients still in continued remission after 18 months. The median duration of response had not been reached yet. Grades 3 or higher CRS and ICANS were 8% and 21%, respectively. This approval set the stage for the first approval for an indolent lymphoma, meeting an unmet need of relapsed refractory indolent lymphomas of which survival in the R/R setting is only 20% at 5 years [5].

## 3 TISAGENLECLEUCEL

Tisa‐cel was first reported in a study of pediatric patients and young adults ≤21 years of age in a phase 2 trial with R/R B‐ALL. A total of 75 patients were treated with tisa‐cel. At 3 months, 81% achieved CR with all patients with a response to therapy having no evidence of measurable residual disease (MRD) by flowcytometry. PFS and OS were 73% and 90% at 6 months, respectively, and 50% and 76% at 12 months, respectively. The median duration of remission had not yet been reached at the time of analysis. Tisa‐cel did have higher incidence of grade 3 or greater CRS at 77% with 48% required therapy with tocilizumab. ICANS was also seen in 40% of patients. Because of the high response rates and overall manageable toxicities, tisa‐cel was approved in 2017 for pediatric and young adults with R/R B‐ALL [6].

For patients with R/R HGBCL, tisa‐cel represented the second approval of CAR‐T cell therapy for this disease when it received FDA approval on August 20, 2017. The basis of this approval was the JULIET study. This study included 93 patients with R/R HGBCL. Lymphodepleting chemotherapy was given in the form of fludarabine and cyclophosphamide or bendamustine. Best ORR was 52% with 40% achieving CR, with response rates seen across all disease risk groups. Toxicities included CRS in 22% of patients and ICANS in 12% of patients. Prolonged cytopenia beyond 4 weeks after tisa‐cell infusion was seen in about one third of patients (32%). No difference in ORR was seen by the level of CD19 expression on tumor cells or expression of immune checkpoint‐related proteins such as PDL‐1, PD‐1, LAG3, or TIM3 [7]. A follow‐up study of 115 patients who received therapy showed an ORR of 53% with 39% achieving a CR. Cytopenias were common adverse events as the most common serious adverse event was CRS (27%). No treatment deaths were reported. These data continue to support that durable activity of tisagenlecleucel shows durable activity and manageable safety profiles, relative to conventional salvage chemotherapy [8].

## 4 LISOCABTAGENE MARALEUCEL

On February 5, 2021, liso‐cel became the third CD19‐targeted CAR‐T product to be FDA approved for R/R HGBCL. This approval was based on the TRANSCEND trial which was a single arm multicenter trial for patients with R/R large B cell lymphoma who had received at least two lines or more of therapy. This study included 192 patients with a reported ORR of 73% with CR rate of 54%. Of the patients who achieved CR, 65% of those patients, or approximately two thirds, had remissions lasting at least 6–9 months. Grades 3 or higher CRS and ICANS occurred in 4% and 12% of patients, respectively. Other significant adverse events included cytopenias and infection [9].

## 5 BREXUCABTAGENE AUTOLEUCEL

Brexu‐cel is the first anti‐CD19 CAR‐T cell therapy approved for/R MCL. In a multicenter single arm study that included 60 patients with R/R MCL who had received a bruton tyrosine kinase (BTK) inhibitor, anthracycline‐ or bendamustine‐based chemotherapy, and CD 20 monoclonal antibody, impressive results were seen in patients who received a single dose of brexu‐cel with reported 93% ORR and 67% CR rate in this study. At 12‐month follow‐up, PFS and OS were 61% and 83%, respectively. Grades 3 or higher CRS and ICANS events were seen at the rate of 15% and 31% respectively; none of which were fatal. As with other CAR‐T studies, additional serious reported adverse events were cytopenias and infections [10]. Despite the high ORR and CR rates with brexu‐cel, data suggest that those patients who relapse after CAR‐T therapy have dismal outcomes [11]. Brexu‐cel was recently approved for adult patients with R/R B‐ALL. This approval was based on ZUMA‐3 which treated 55 patients with brexu‐cel. At the median follow up of 16.4 months, CR/CRi rate was 71% with MRD negativity in 97% of responders. Median duration of remission was 12.8 months, and median OS was 18.2 months [12].

### 5.1 MM

#### 5.1.1 Idecabtagene vicleucel

The first and currently only CAR‐T cell therapy available for treatment of MM is idecabtagene vicleucel (ide‐cel) which is a B‐cell maturation (BCMA)‐directed CAR‐T product. It received accelerated FDA approval on March 29, 2021 for patients with R/R MM who have received four or more lines including a proteosome inhibitor, an immunomodulatory agent, and an anti‐CD38 monoclonal antibody. Of 128 evaluable patients in the KarMMa study, ORRs were 73% with CR rate of 33%. PFS and OS were 8.8 and 19.4 months, respectively. MRD negativity was confirmed in 79% of patients who achieved a CR or better. Grade 3 or higher CRS was low at 5%, and overall ICANS was seen in 3% of patients with no higher than grade 3 ICANS. Overall, the data showed that heavily treated patients with R/R MM who received ide‐cel shows response with over a quarter of patients achieving MRD status [13]. In a retrospective study that compared real world outcomes of patients who received available SOC therapies with outcomes of the KarMMa study, ide‐cel significantly improved responses, PFS, OS compared to currently available therapies in triple class exposed R/R MM [14].

#### 5.1.2 Ongoing clinical trials

The field of CAR‐T therapy is rapidly changing with many ongoing clinical trials in various hematological malignancies. These studies can be broadly divided into three categories. (1) Studies examining the role of currently approved CAR‐T products in different clinical settings. (2) Novel CAR‐T therapies targeting same or different tumor‐associated antigens. (3) And, Studies testing the combinations of CAR‐T with various drugs to improve safety and efficacy of CAR‐T therapy.

The currently approved CAR‐T therapies are available for patients with R/R HGBCL or FL after 2 or more lines of therapies. Brexu‐cel is approved for R/R MCL; however its role in BTK‐naïve MCL is still under investigation (ZUMA 2, cohort 3, NCT04880434) to fulfill FDA postmarketing requirement. ZUMA‐12 (NCT03761056) is testing the safety and efficacy of axi‐cel as a first‐line therapy in patients with high risk HGBCL. Patients with positive interim PET scan after two cycles of chemoimmunotherapy received axi‐cel. In a planned interim analysis of 15 response‐evaluable patients, ORR and CR were 93% and 80%, respectively [15]. The current SOC for relapsed HGBCL after first line chemoimmunotherapy is salvage therapy followed by consolidative autologous hematopoietic cell transplantation (auto‐HCT). ZUMA‐7 (NCT03391466) was a randomized phase 3 study which compared axi‐cel versus salvage therapy followed by auto‐HCT. The study has met its primary endpoint showing significantly better EFS and ORR with axi‐cel compared to SOC therapy; however, final publication is pending [16]. Similar results have been announced using liso‐cel is a second line therapy for HGBCL in another phase 3 study (TRANSFORM, NCT03575351) [17]. Contrary to these two trials, a comparable phase 3 trial (BELINDA, NCT03570892) using tisa‐cel failed to meet the primary endpoint [18]. These findings highlight the challenges associated with doing comparisons across the studies utilizing different CAR‐T cell products in the same clinical setting. Tisa‐cel is being tested in newly diagnosed B‐ALL with persistent end of consolidation MRD after first‐line therapy in pediatric and young adult patients (CASSIOPEIA, NCT03876769). Given encouraging results with Ide‐cel in R/R MM, KarMMa‐4 is testing safety and efficacy of ide‐cel in newly diagnosed high risk MM [19].

Advances in genetic technologies have led to the development of CAR‐T therapies targeting novel antigens across different malignancies [20]. The future generations of CAR‐T therapies have potential to salvage the patients who have failed currently available CAR‐T therapies. Multi‐antigen targeted CAR‐T therapy may overcome the issue of antigen‐escape relapses after single antigen‐directed CAR‐T therapy. CAR‐T therapies targeting CD123 and NKG2D have shown some efficacy in relapsed acute myeloid leukemia (AML) [21, 22]. In a phase I/II study, CD30‐directed CAR‐T‐cell therapy showed ORR of 72% and CR rate of 59% in R/R Hodgkin lymphoma with an excellent safety profile [23]. T cell malignancies are inherently difficult to target with CAR‐T‐cell therapy due to morbidity associated with prolonged T cell aplasia and limited in vivo expansion due to fratricide. Multiple ongoing studies are testing safety and efficacy of CD5, CD7, CD4 ‐directed CAR‐T in R/R T cell lymphoma, and T cell ALL. In a phase I study, Pan et al. showed high CR rate with manageable safety profile of donor‐derived CD7 CAR‐T cell in R/R T‐ALL [24].

Multiple studies are testing the safety and efficacy of CAR‐T‐cell therapy combined with various immunomodulatory agents in different settings to improve in vivo persistence/extension of CAR‐T cells. Gauthier et al. has shown safety of concurrent ibrutinib (BTK inhibitor) with CD19 CAR‐T‐cell in relapse/refractory chronic lymphocytic leukemia. This approach led to higher rates of MRD clearance compared to CAR‐T‐cell therapy alone [25]. Similar approach has been described in patients with R/R NHL, where repeat infusion of CD19 CAR‐T‐ cell therapy after pretreatment with ibrutinib led to better in vivo expansion of CAR‐T cells and disease responses [26]. The preliminary analysis of ZUMA‐6 (NCT02926833) showed that the combination of atezolizumab (anti‐PD‐L1 monoclonal antibody) with axi‐cel was safe, but ORR and CR were comparable to single agent axi‐cel in patients with R/R HGBCL [27]. In MM space, multiple studies are ongoing combining BCMA‐CAR‐T with IMiD or anti‐CD38 monoclonal antibody [28].

#### 5.1.3 Impact of CART on clinical practice

CART has had significant impact and changed the paradigm for the treatment of large B cell lymphoma, FL, marginal zone lymphoma, MCL, and ALL [9, 29, 30].

Patients with relapse or refractory HGBCL have a particularly poor prognosis as the likelihood of response to salvage chemotherapy is 26%, and medial OS is 6 months. Many of these patients end up going from one toxic chemotherapy to another and getting sicker and sicker until they go on palliative care or hospice. However, with the advent of axi‐cel, this same patient population now has an ORR of 84%, CR rate of 59%, and median OS at 2 years not reached [3]. Tisa‐cel also had high response rate of 52% and 1‐year relapse‐free survival of 65% [30]. Taken together, CARTs have extended the life of patients with NHL and made great impact in the clinical management of HGBCL [31].

The previous standard was to funnel patients with relapse HGBCL to salvage chemotherapy followed by auto‐HCT. Some of the expected toxicities include neutropenic fever or other infection, mucositis to name a few, and physicians know how to look for those symptoms and signs. CART has changed this paradigm and introduced new pattern of toxicity bringing with it comes terminology that is new to the internist such as: CRS, ICANS, prolonged cytopenia, and hypogammaglobulinemia. Physicians need to rapidly learn the terminology, how to grade, and what to do at each point in the care of patients. Physicians at the academic centers need to know these terminologies and teach their staff as well as referring physician practices. Also, whereas many chemotherapy infusion clinics are closed in the after‐hours and weekend, patients who have received CARTs can become critically ill at any time; therefore, many centers are changing the pattern of staffing to allow rapid triage and treatment of these patients.

Unlike chemotherapy that is given as multiple cycles over many months, CART is a single infusion in most cases. In HGBCL, responders to CART do not need maintenance therapy. This allows them to spend more time away from oncology and more time in the community. Practices will adjust to this new follow‐up pattern.

CART have changed the pattern of clinical practice in that we are having much fewer referrals to hospice and end of life care because more patients who received CARTs achieve complete and durable response compared to chemotherapy alone. CART patients use hospital bed earlier in the treatment process during the active phase of monitoring for and treating higher grade acute toxicities including CRS and neurological complications. Hospital bed use after this time is rare. High‐volume CAR‐T centers are developing expertise to manage CAR‐T‐treated patients in the outpatient settings. This will allow significant cost‐savings and better quality of life.

In summary, CART therapy is new and during the past few years has become firmly embedded in the treatment of HGBCL, FL, MZL, and B‐ALL. As it is becoming more popular and able to be used in earlier lines of therapy, the paradigm and our practice pattern will continue to adapt to this new reality. As the safety profile improves and CARTs able to be given in centers that are not necessarily tertiary centers of excellence, practice patterns will continue to change to manage new toxicities and accommodate the improved survival across the board.

## CONFLICT OF INTEREST

The authors declare that there is no conflict of interest that could be perceived as prejudicing the impartiality of the research reported.
